# The Charity Organisation Society and the Treatment of Tuberculosis in London

**Published:** 1914-07-18

**Authors:** 


### THE CHARITY ORGANISATION SOCIETY AND THE TREATMENT OF TUBERCULOSIS IN LONDON.

Long amongst the most ardent supporters of the dispensary system in the treatment of tuberculosis, the Charity Organisation Society initiated in the spring of this year a comprehensive inquiry concerning the arrangements which have so far been made in London for the prevention and treatment of the disease. The Society fairly claims to occupy the position of the outsider, identified with no one section either of medical or social opinion; and the report of the inquiry demands the serious attention of all those interested in tuberculosis work.

The report, while emphasising the difficulties of working a tuberculosis scheme for an area so immense and so populous, and making due allowance for the complexities necessarily arising as the result of divided authority, points out clearly the lack of cohesion, the confusion and misunderstanding between the various authorities in London.

The Insurance Committee for the County of London, hampered by lack of institutional accommodation, has made shift to deal with a large number of cases by machinery consisting of panel doctors and a medical referee, and by using a number of beds in London hospitals, in sanatoriums, and in other institutions—a number utterly inadequate for the demand. The difficulty arising from a flaw in the Act by which the Committee is debarred from using beds in Poor-Law institutions (those of the Metropolitan Asylums Board being technically included) has been, as is well known, partially evaded as regards the hospitals of that Board. The Committee "has had to make use of buildings not altogether suitable for its purpose, and has sent to the same institution patients in all stages of the disease, conditions which make effective treatment impossible. The patient is frequently discharged too soon to have materially benefited. It is to be deplored, also, that, as a general rule, no attempt is made to keep in touch with him on his discharge," so that, even if progress has been made, "there is little prospect of it being maintained."

"Insured patients, at the present time, have no proper choice of institution open to them." The Society wishes to draw special attention, as it has frequently done before, to the lack of accommodation for cases of advanced disease; whilst there is little hope of curing the individual patients, there is an urgent need for the protection from infection of those who surround them. A sufficient provision of suitable institutions for such cases would prove a most valuable preventive measure; "if properly equipped, and within fairly easy reach of the patient's home, persuasion would suffice to secure the patient's entry, and compulsory powers of removal, which would almost inevitably lead to concealment of the disease, need not be invoked." The Charity Organisation Society has always experienced the utmost difficulty in securing accommodation anywhere for this type of case. The difficulties of the Insurance Committee, with its far larger number of cases, are still greater. The great majority of insured persons who are in an advanced stage of consumption must, at present, either remain at home or enter an infirmary.

With regard to the provision of sanatoriums, hospitals, and other institutions for London patients very little progress has as yet been made. The second unit of the dispensary scheme is altogether incomplete, and it cannot be claimed that a sufficient selection of appropriate institutions is yet available even for insured persons. Patients in varying stages of the disease are being sent to the same institution. The general provision by public authority of institutional treatment for the uninsured who suffer from tuberculosis has not yet been undertaken. "To summarise the matter, it may be said that, so far as insured persons are concerned, it has not yet been found possible to organise the available accommodation under the scheme; as to the uninsured, no new accommodation at all has yet been provided."

The report states very clearly the case for the dispensary system of treatment and the arguments and representative opinions which have led the Society to its advocacy of that system. Whilst, in cases of tuberculosis in the early stage, the difficulties of diagnosis may be considerable and, consequently, the views of a hospital consultant are of very great value, the Society's many years' experience of dealing with tuberculosis among working-people has convinced it that no measure of attack upon the disease has any chance of success unless it begins and ends with the home, and includes both the continuous care of those found to be tuberculous, and the examination and supervision of contacts. A dispensary is established largely (some would say primarily) for purposes of prevention. The greater number of indefinite or suspected cases are to be found among children. "The history of the past four months, however, shows that there has been some tendency among hospital authorities to propose schemes under which the hospital dispensary will consist of little more than an out-patient department under a new name."

The Charity Organisation Society refrains from comment at length upon the history of twelve months' effort to deal with tuberculosis in London. "The whole course of events illustrates in a remarkable way the danger and difficulties involved by divided authority." Looking back upon these events, the report carefully deals with the methods of the tuberculosis dispensaries, and adequately weighs the opinions of those in charge of voluntary dispensaries, and of medical officers of health, hospital physicians, and others. The Society is, whilst in favour of the dispensary as a centre of organisation, "convinced that the eventual eradication of tuberculosis (in London as elsewhere) depends not upon any one type of institution or of treatment, . . . but upon a combination of methods and a conjunction of forces."

